# Policy environment and male circumcision for HIV prevention: Findings from a situation analysis study in Tanzania

**DOI:** 10.1186/1471-2458-11-506

**Published:** 2011-06-28

**Authors:** Joseph R Mwanga, Mwita Wambura, Jacklin F Mosha, Gerry Mshana, Frank Mosha, John Changalucha

**Affiliations:** 1 National Institute for Medical Research, P.O. Box 1462, Mwanza, Tanzania

**Keywords:** Medically performed male circumcision, HIV infection, AIDS, policy environment, Tanzania

## Abstract

**Background:**

Male circumcision (MC) has been shown to be effective against heterosexual acquisition of HIV infection and is being scaled up as an additional strategy against HIV in several countries of Africa. However, the policy environment (whether to formulate new specific policy on MC or adapts the existing ones); and the role of various stakeholders in the MC scale up process in Tanzania was unclear. We conducted this study as part of a situation analysis to understand the attitudes of policy makers and other key community and health authority decision makers towards MC, policy and regulatory environment, and the readiness of a health system to accommodate scaling up of MC services.

**Methods:**

We conducted 36 key informants' interviews with a broad range of informants including civil servants, religious leaders, cultural and traditional gatekeepers and other potential informants. Study informants were selected at the national level, regional, district and community levels to represent both traditionally circumcising and non-circumcising communities.

**Results:**

Study informants had positive attitudes and strong beliefs towards MC. Key informants in traditionally non-circumcising districts were willing to take their sons for medically performed MC. Religious leaders and traditional gatekeepers supported MC as it has been enshrined in their holy scripts and traditional customs respectively. Civil servants highlighted the need for existence of enabling policy and regulatory environment in the form of laws, regulations and guidelines that will ensure voluntary accessibility, acceptability, quality and safety for those in need of MC services. Majority of informants urged the government to make improvements in the health system at all levels to ensure availability of adequate trained personnel, infrastructure, equipment, and supplies for MC scale up, and insisted on the involvement of different MC stakeholders as key components in effective roll out of medically performed MC programme in the country.

**Conclusions:**

Findings from the situation analysis in Tanzania have shown that despite the absence of a specific policy on MC, basic elements of enabling policy environment at national, regional, district and community levels are in place for the implementation of MC scale up programme.

## Background

The relationship between medically performed male circumcision (MC) and HIV is crucial, because three randomised controlled trials conducted in South Africa [1], Kenya [2] and Uganda [3] have demonstrated that MC reduces the risk of heterosexual HIV infection by approximately 60%. The overall prevalence of MC in Tanzania is estimated at 70% with regional variations from 26.4% in Kagera to 97.9% in Dar es Salaam. In 4 out of 5 regions with the HIV prevalence above 6%, MC prevalence is at or below 43% [4]. In March 2007 the World Health Organisation (WHO) and the United Nations Joint Programme on AIDS (UNAIDS) recommended that MC should be considered as part of a comprehensive HIV prevention package [5]. In the Eastern and Southern Africa (ESA) sub-region, 13 countries including Tanzania with high HIV prevalence, low levels of MC and generalised heterosexual epidemics were identified as priority countries for MC scale up [6].

The government of Tanzania is keen to undertake MC on a large scale. However, there was a dearth of information to inform the policy development process for scaling up of MC. We conducted situation analysis to find out whether there is an enabling policy environment that supports MC scale up at the national level, regional, district and community levels. We specifically investigated: (i) attitudes and beliefs of informants concerning MC; (ii) the policy and regulatory environment to determine what relevant policies and regulations exist and how they might influence decisions on MC; and (iii) the readiness of the health system to support the scaling up of MC services.

Two policies exist in Tanzania: The National HIV/AIDS Policy of 2002 and the Traditional and Alternative Medicine Act of 2002. However, the former policy does not provide for MC whereas the latter does not regulate traditional MC practices. Furthermore, there are few key national policy documents which promote and recommend scaling up of safe MC as one of the new HIV prevention interventions: The National Multi-sectoral HIV Prevention Strategy (2009-2012) [7] has one of its strategic priorities as the promotion of safer sexual behaviours and reduction in risk taking behaviours through the introduction and targeted scale up of MC. The Health Sector HIV and AIDS Strategic Plan II (2008-2012) [8] notes that MC has not been integrated in current HIV prevention services and has as one of the strategic objectives the promotion of medically performed safe, acceptable MC for health benefits and as a preventive measure against HIV transmission.

The relevant policies governing basic public health and surgical services should support the provision of comprehensive MC services of high quality in a way that will make them widely accessible and available [9]. This would include policies governing who can provide minor surgical procedures under local anaesthesia, the level of the health system at which they can be performed and under whose authority, as well as policies concerning quality control and aspects of medical ethics, e.g. voluntary consent, age of consent and decision-making by parents and guardians on behalf of children. It would extend to legal and regulatory issues concerning traditional health practices and practitioners. There may be legal and regulatory issues to investigate with respect to necessary commodities, e.g. if surgical MC kits are going to be produced or if specific medical devices are to be imported or manufactured. However, this policy and regulatory framework is not in place in Tanzania and therefore it is necessary to focus efforts on either revising specific elements of existing policies so as to address the gaps, or, introducing a specific new policy. In this article the term 'policy' means a group of decisions and guidelines for the implementation of a particular issue, in this context, MC.

## Methods

Between 2008 and 2009 we conducted a situation analysis of MC in Dar es Salaam City, one traditionally circumcising district (Tarime district in Mara region) and two traditionally non-circumcising districts (Bukoba district in Kagera region and Ileje district in Mbeya region). Prior to data collection, investigators visited all the study sites and made appointments for interviews. The purpose of the interviews was to obtain detailed information about attitudes, beliefs and practices regarding circumcision and the policy environment from the perspective of the informants. Data were collected through key informants' interviews (KIIs) which were semi-structured and consisted of open-ended questions.

Thirty six key informants (KIs) were purposively selected from a diverse range of people whom we thought could provide us with the detailed information, on the basis of their expertise or knowledge of MC. These are people who due to their special position, are looked upon as representatives of the opinions and experience of the community. These KIs were likely to have knowledge or experience that is relevant or important to a circumcision programme or represent important stakeholders (those who are able to affect the programme and those who will be affected by it).

The KIs were from community, district, regional and national levels. The national level KIs included the permanent secretary, the chief medical officer, the director of hospital services at the Ministry of Health and Social Welfare (MoHSW) and other directors and programme managers of institutions related to health services. At the regional, district and community levels; KIs included politicians, heads of health service departments, religious leaders of all faith and denominations and traditional and cultural gatekeepers such as tribal/clan leaders, traditional circumcisers and traditional healers. Leaders of non-political and non-religious youth and women groups, and directors of local non-governmental organisations (NGOs) dealing with HIV prevention and leaders of people living with AIDS were also included. The KIs were not screened for circumcision status. For the sake of safeguarding identities of our informants, some of potential identifiers will not be given at the end of quotations. Four trained research assistants, (two females and two males), were involved in conducting the same sex interviews in *Kiswahili*, the national language. The interviews were tape-recorded and noted down manually.

Data were transcribed verbatim and translated into English. Each transcript was examined line-by-line using QSR NVivo 2.0 qualitative software (Pty Ltd, Sydney, Australia). Texts were coded using a coding scheme that was developed prior to coding. Emerging theme codes were also added as they appeared and at regular intervals, major theme categories were reviewed to ensure that classification was adequate. Thematic coding allowed two social scientists (JRM and GM) to undertake a grounded analysis. This inductive approach was used in order to allow the data to direct the progression of the intended and emergent themes. Analysis of data used explicit, systematic and reproducible methods. Hence, validation and trustworthiness of the findings was established [10].

Ethical clearance for this study was obtained from the Tanzanian Medical Research Co-ordinating Committee. The study was also approved by Columbia University ethics Committee and the ethics committee of the Centres for Disease Control and Prevention (CDC) and Global AIDS Programme Associate Director of Science (GAP/ADS). An information sheet describing the study objectives, procedures, risks and benefits was read to study participants and informed consent was sought through a signed/thumb printed consent form.

## Results

### Socio-demographic characteristics of informants

Thirty six KIs were interviewed: 11 in Mara, 10 in Kagera, 9 in Mbeya and 6 in Dar es Salaam regions respectively. Of these; 34 (94%) were males, 26 (72%) were aged between 40 and 59 years, 32 (89%) were married and 21 (58%) had diploma, degree and postgraduate degree education. Majority of informants 30 (83%) were Christians and 22 (61%) were civil servants. The dominant ethnic groups in the study communities (districts) apart from Dar es Salaam, the *Kurya, Haya *and *Nyakyusa *were slightly more than half 19 (53%) of the KIs; whereas, 17 (47%) of the KIs belonged to other ethnic groups found in Tanzania.

### Attitudes and beliefs towards MC

Most of KIs 34 (94%) was aware of the availability of MC services at the health facilities in their areas including the district hospitals. For example, in Ileje district majority of informants said that circumcision services are available at Isoko mission hospital and Itumba health facility. Some informants in Bukoba district reported that circumcision services are available at the distant district hospital in Izimbya resulting in a few people in that area opting to go for medically performed MC.

Male informants in Bukoba and Ileje districts (traditionally non-circumcising districts) generally supported medically performed circumcision considering it to be safer, more hygienic and the wound heals faster than circumcision performed by traditional circumcisers. One KI remarked: "*For the sake of better wound treatment, pain reducing drugs, less blood loss and anaesthesia, I would rather take my son for circumcision at a health facility." *[KI, 30 years old male leader of youth group from Ileje district, Mbeya region].

Almost all KIs 35 (97%) showed willingness to support the scale up of MC services. They were enthusiastic to take part in sensitisation and health education campaigns and in performing circumcision procedures if they are asked and empowered to do so. One KI remarked: *"Our institution will make sure that our community will be sensitised to undergo medically performed MC. I will use my position in the Ward and District Council to make sure that circumcision is being performed widely in our hospitals and dispensaries." *[KI, 49 years old male civil servant from Tarime district, Mara region].

In order to increase demand for circumcision services, all KIs suggested that the services must be affordable. If the services should be paid for, majority of KIs 34 (94%) said that the amount shouldn't exceed 5,000/ = Tshs. (roughly 3US$) per person. Otherwise they suggested that the government should provided MC services free of charge due to prevailing economic hardships facing many Tanzanians particularly those living in rural areas with no reliable sources of income. Alternatively payment in kind (equivalent of the cost of farm produce) was also suggested.

Other factors influencing MC mentioned by the majority of informants 34 (94%) were broadly categorised into 3 groups: those related to culture, health, and religion. With regard to culture, almost all informants echoed the adherence to traditional customs as the most important factor which influence people to undergo circumcision in traditionally circumcising communities such as the *Kurya *of Mara region. This view was explicit in almost all interviews. Circumcision is performed in traditionally circumcising communities as a rite of passage into manhood from time immemorial.

Religion was mentioned to influence circumcision as well. It was reported that both Muslims and Christians are required by their religious faiths to get circumcised so as to adhere to religious purity. Despite of having a few Muslims in our study sample 4 (11%), it was pointed out by many informants, predominantly Christians, from traditionally non-circumcising communities that Islamic faith insists on its believers to get circumcised. It is '*suna*'(commendable) and a religious requirement as a 'civilised' man. One religious leader said:*"Circumcision is a religious order from our leader Prophet Mohamed S.A.W. who taught us that if you are not circumcised, urine may be retained in the foreskin, and even during preparations for praying it is difficult to be thoroughly clean hence you become short of 'udhu' (religious ablution)."*[KI, 63 years old male, religious leader from Bukoba municipality, Kagera region].

In the same vein, another religious leader said:*"In religious circles there is what is called 'purification,' that is the relationship between man and his creator. If you undergo circumcision then you maintain relationship with God and your clan. You become part of them. Second, in Christian faith there are many places in the Holy Bible that underscores the importance of circumcision. In Israel, circumcision was regarded as one of the cornerstone of religious faith, and if one is not circumcised he was regarded in Kiswahili as 'mtu wa mataifa' (non-Jew) and is not the son of God. In Catholic faith, circumcision does not end up with cleanliness of the body but of the soul too. Therefore, for Catholics circumcision donates relationship between a person (a Catholic) and God. Circumcision is an upbringing religious wise, health wise and culturally." *[KI, 52 years old male, religious leader from Musoma municipality, Mara region].

Nevertheless, circumcision was not a common practice in traditionally non-circumcising communities of Kagera and Mbeya regions because it was not part of their culture. However, with the influence of the so called 'forces of modernity' (e.g. schooling, religion, modern health care, and exposure to other cultures), traditionally non-circumcising communities are increasingly accepting MC. One KI observed:*"For those who had an opportunity to go out of Ileje district, to Dar es Salaam for instance, they have to get circumcised so as win girl friends and lovers. But while in Ileje, they don't bother to get circumcised because according to traditions and customs from time immemorial it is a taboo for a man to get circumcised"*[KI, 35 years old male, traditional healer from Ileje district, Mbeya region].

### Health system readiness

#### Enabling policy and regulatory environment

The legal, regulatory and policy framework governing MC in Tanzania is weak and there is no law dealing specifically with circumcision. MC, as one of HIV infection risk reduction strategies, is governed by general laws that regulate the medical profession and the provision of medical services in medical settings by health professionals. In the absence of such a specific legal and regulatory framework on MC one KI made the following suggestions: *"Tanzania Medical Council should develop specific ethical guidelines to assist health workers to understand their duties in relation to MC; to review the existing legal and regulatory framework, including relevant customary laws, action plans and strategy papers and practices; convene community consultations with a broad range of relevant stakeholders, including technical experts from the National AIDS Control Programme, traditional providers of MC (in regions where MC is an existing practice), representatives of human rights and legal institutions/organisations, women's groups, men's groups working towards gender equality, youth groups and other civil society groups so as to develop specific policies and laws relating to MC to ensure accessibility and safe provision and with sufficient safeguards for all stakeholders" *[KI, 57 years old male civil servant from Dar es Salaam City].

Therefore, there still remains lack of clarity on laws that will ensure medically performed MC services are accessible, provided safely and with sufficient safeguards for all clients. One KI remarked: *"For a long time, MC services have been provided for those in need in the health care delivery system as well as in communities by traditional circumcisers as private arrangements between the clientele and the health care providers. MC has not been officially recognised as a routine service for health benefits, let alone for HIV prevention."*[KI, 79 years old male, former traditional leader from Ileje district, Mbeya region].

Appropriate by laws and regulations are needed so that MC services are accessible and provided safely without discrimination and that different stakeholders are guided and protected by law. Underscoring the importance of enabling policy environment for MC scale up one KI at the national level expressed his views as follows:*" The existing policy environment may shape decisions on how to address the national strategy as well as decisions on the clinical protocols and guidelines. Elements of the national strategy for scale up (e.g., the desired level of services or types of service providers) may in turn highlight policy gaps and drive efforts to address them." *[KI, 54 years old male civil servant from Dar es Salaam City].

Nevertheless, the protection and promotion of human rights is integral to all aspects of HIV prevention, treatment, care and support. One KI made a following comment: "*The initiation and expansion of MC services must ensure that the procedure is carried out safely, under conditions of informed consent and without discrimination" *[KI, 43 years old male leader of people living with HIV/AIDS from Tarime district, Mara region].

#### Investments in health facilities, human resources, equipment and supply of MC materials

The most common issues related to the supply of services pointed out by study informants in a bid to increase the provision of MC were related to availability of: adequate health facilities, human resources, equipment, and materials. One KI stressed that: "*The government should budget and ensure adequate funds available for laying down infrastructure, purchase of modern major and minor equipment, drugs, supplies and adequate qualified staff. There should be enough health facilities both private and public dispensaries, health centres and hospitals providing circumcision services to increase accessibility. Therefore, there is a need to strengthen the entire health system before scaling up." *[KI, 42 years old male civil servant from Bukoba municipality, Kagera region].

Similarly, another KI added: *"The health sector in Tanzania has a shortage of human resources and other necessary financial and material resources for MC scale up. The availability of trained human resources, necessary commodities, existing underlying elements of quality (counsellors/counselling skills and infection prevention practices); space, medicine, supplies and equipment, record keeping and information systems, adequate follow-up, all should be investigated to see what investments may be required to introduce safe, quality comprehensive MC services at different levels of the health system. These will also help to determine whether efforts at task shifting are required, what types and level of training are needed to develop the necessary human resources. It will also inform decisions about how to design and integrate appropriate supervision and quality improvements, health management information system and monitoring and evaluation efforts." *[KI, 65 years old male civil servant from Mbeya City, Mbeya region].

#### Integrating medically performed MC into existing health care delivery system

The key informants particularly those at the national level also supported MC scale up but had reservations regarding the mode of integration. One KI commented: "*Male circumcision should be regarded as an ordinary surgical procedure which has been into existence since long time ago. It should be formalised. Scale up should learn from the current practice and experiences to enhance acceptability and sustainability. If it is regarded as a new intervention it may push up the costs. However, the formalisation should be done with caution as it may bring conflict of interest among facility-based health care providers who are currently earning money from performing the procedure." *[KI, 57 years old male civil servant from Dar es Salaam City]. Furthermore, in a bid to integrate MC services into other health services one KI suggested that*:" Infant circumcision for instance, should be integrated into reproductive and child health (RCH) services and provided for free" *[KI, 42 years old male politician from Bukoba district, Kagera region].

More importantly, Tanzania has been implementing HIV prevention programmes since the 1980s; the scaling up of MC services will not replace the already known effective prevention methods but rather complement them. For effective integration of MC for HIV prevention into existing HIV prevention, reproductive health and other regular health services, one KI observed: *"The success of MC for HIV prevention depends on the effectiveness of the directorate of hospital services and overall coordination by the MoHSW. The directorate should coordinate the line departments, in particular prevention and treatment of sexually transmitted infections, reproductive health services, surgery, laboratory and HIV and AIDS prevention. Male circumcision for HIV prevention services should not only be an integral part of hospital services but any up scaling should be towards improving the quality and access to these services, in particular for adolescents and young adult males." *[KI, 55 years old male civil servant from Tarime district, Mara region].

#### Integrating traditional MC practices into existing health care delivery system

Tied to the integration of medically performed MC into existing health care delivery system is the integration of traditional MC practices into the existing health care delivery system. The majority of informants 34 (94%) pointed out the need to integrate the services of traditional male circumcisers into hospital services due to complications in the traditional circumcision set-up. However, conflict of interest may also arise if in the course of scaling up circumcision services, traditional circumcisers and clan leaders are not taken on board. One KI remarked: "*Among the Kurya of Tarime district traditional circumcisers receive payments (in kind or money) for circumcision. This payment is shared between traditional circumcisers and clan leaders (wazee wa ukoo). Clan leaders command respect and power in their communities; therefore, if the integration would result into loss of their income and respect, then there is a danger of resistance to efforts to scale up medically performed MC*. *To avoid such a possible conflict of interest, clan leaders and circumcisers should be engaged from the very beginning and throughout the process through dialogue in campaigns for scaling up of MC." *[KI, 52 years old male leader of local NGO from Tarime district, Mara region].

Another KI advocated for close cooperation between traditional and hospital based circumcisers as follows: *"I urge health care providers to work closely with traditional circumcisers (locally known as Omusari in Kurya language) because they together with clan leaders are the custodian of Kurya traditions and chief spokesmen and decision makers on when and who has to undergo circumcision" *[KI, 51 years old male traditional circumciser from Tarime district, Mara region].

#### Quality assurance of MC services

Regarding issues related to quality of MC services a concern was raised by a minority of KIs 6 (17%) if the operation is done in traditional settings where the possibility of sharing surgical instruments without proper sterilisation is high, may lead to acquisition of infections such as tetanus and even HIV. Furthermore, if the procedure is done by an incompetent traditional circumciser, there is chance of disfigurement of the penis, excessive bleeding, and even death may ensue. Similar risks were also anticipated in medical settings if the procedure is done by unqualified medical personnel.

#### Information, communication and advocacy for MC scale-up

Responding to the question on how to increase demand for MC, almost all 35 (97%) of the KIs mentioned advocacy and mass education as critical issues. With regard to education, it was pointed out that advantages and disadvantages of medically performed MC need to be communicated to the people to raise their levels of knowledge and awareness on the issue and to dispel fears, misconceptions and stigma. The most common approach mentioned was to educate people through public meetings and other such gatherings. One KI stressed: *"Health education campaigns for MC scale up should be preceded by advocacy. I urge politicians not to rush into sensitising the public before health care system is ready to absorb the demand for MC and the whole process should not be politicised, but rather taken as a routine procedure and do away with bureaucracy."*[KI, 78 years old female leader of Women's group from Bukoba district, Kagera region].

In support of advocacy and awareness campaigns, one KI made the following comments: *"Influential people should be involved. These would include leaders of political parties, government leaders and civil servants from national to community levels, health staff and religious leaders. Others are influential and respected people in the communities including traditional leaders (such as former chiefs and clan leaders), circumcisers and healers, teachers at primary and secondary schools, peers and parents. All these need to be well informed of the intended campaign through workshops and in turn will use various platforms to mobilise people for increased demand for MC." *[KI, 56 years old female civil servant from Musoma municipality, Mara region].

## Discussion

### Attitudes and beliefs towards MC

The perceptions of acceptability of MC by different groups including potential clients (target population), family members, partners, spouses, parents, service providers and other influential people is vital. It is important to know people's beliefs and understanding about MC with a view to informing policy and strategic and operational plans. The socio-cultural aspects of MC, particularly those surrounding any current or historical traditional circumcision practices, are also important in determining effective strategies for either attempting to modify the practices or for taking advantage of them. Findings of this study revealed high levels of acceptability and strong beliefs in medically performed MC in both traditionally and non-traditionally circumcising communities. The support for medically performed MC in communities which undertake traditional MC has been also reported in other studies in Tanzania [11,12]. More importantly; people who could influence policy, delivery, and uptake of MC at different levels of government and health system were also supportive.

Cost for circumcision is one of key elements of a national MC policy [9]. The maximum cost of 3US$ for medically performed MC services that informants in this study were willing to pay is similar to the median cost for circumcision in a traditional settings reported in another study in Tanzania [12]. Determining the cost of MC services may be influenced by existing policy, depending on whether MC is seen as part of a basic health care package or whether it is priced in line with other minor outpatient services or other preventive services. The mix of public and private services also affects both cost and accessibility.

### Enabling policy and regulatory environment

Although it has been some years since the protective effect of medically performed MC was confirmed, there is no policy that regulates provision of circumcision services in the health care delivery facilities in Tanzania. However, since June 2010, there has been a two years (2010-2012) national strategy for scaling up MC for HIV prevention which is intended to serve as the main guiding document for MC practices and services at all levels of the health delivery system in Tanzania [13]. The said document is a product of this study together with the other two nested studies [11,12]. Thus, the scale up strategy was based on sound scientific evidence. However, one could argue that relevant technical policies and guidelines for MC scale up are of crucial importance. Enabling policy and regulatory environment is one of ten key elements which have been identified as critical to MC programme scale up [9].

On the one hand, there are varying views on the necessity of having a specific policy on MC for HIV prevention. In the recent Eastern and Southern Africa sub-regional consultation, some countries felt that it was a necessary process to have a stand-alone policy to ensure appropriate mobilisation of resources to enable rapid scale up. As of end of December 2009 MC policy was in place in four countries of Kenya, Lesotho, Swaziland and Zimbabwe [6].

It should be noted that the provision of comprehensive MC services involves policy, regulatory and human right issues. A policy, regulatory or legal framework should be in place to govern medical ethics, for example confidentiality, discrimination and stigmatisation, provision of information, voluntary consent, age of consent, and other human rights considerations. The policy may also cover issues of quality assurance, for example the certification/accreditation of facilities and providers in public and/or private sectors, processes for investigating of abuse and recourse or procedure in case of malpractice [9].

However, the primary duty of formulating and implementing laws and regulations governing the provision of medically performed MC services is vested in national governments. Countries should ensure that it is provided with full adherence to medical ethics and human right principles; informed consent, confidentiality and absence of coercion [14]. The national MC policy should establish the overall goal and outline the guiding principles of the programme and will be reflected in the national strategy. The key elements of a national MC policy have been summarised in the WHO & UNAIDS publication [9].

Continuing with the debate on whether to have a specific policy on MC or not, some countries felt that formulation and approval of a separate policy for MC was time consuming process, and could potentially create barriers for rapid implementation; as a result they preferred to encapsulate MC in existing HIV prevention policy, Tanzania is a case in point. Therefore, it is critical to understand that a new dedicated policy on MC is often not required or necessary, as long as the basic elements of an enabling environment must be in place to allow the scale up of accessible and acceptable MC services. Even if the relevant policy environment exists, however, there are other important steps that should be taken to clarify that and the 'official' position on the provision of MC services. The most crucial contextual factor in this case is the political goodwill of the President of the United Republic of Tanzania who is keen for MC to be introduced as an additional strategy against HIV infection.

### Health system readiness

It is critical to determine the readiness of the health system to support the scaling up of MC. As a relatively simple outpatient surgical procedure, many levels of formal health systems may be able to provide medically performed MC services with little additional investments. What is needed is the understanding of the context in which services can be offered, which will help inform strategic decisions around where to prioritise investments in human resource, equipment and infrastructure.

However, the development and expansion of MC services for HIV prevention should not disrupt health systems and the implementation of other health programmes. In this study participants highlighted the need to improve the health system at all levels to ensure that the demand created for MC services will be adequately met. This requirement is in line with WHO and UNAIDS's key policy and programme recommendation number 8 which urges for the need to strengthen health services to increase access to safe MC services [5].

In order to scale up MC for HIV prevention one of the strategic actions which has been undertaken by the government of Tanzania is coordination, leadership and management of the process. The WHO supported the Tanzanian MoHSW to form two bodies (the taskforce committee and technical working group) to oversee the scaling up of MC services in the country. These bodies were formed through a consultative and inclusive process involving all stakeholders. Members of the oversight bodies were selected based on among other criteria: experience in policy formulation, implementation and advocacy issues; and technical competence in circumcision [15].

Among the decisions taken by the two oversight bodies were designating a focal person at the MoHSW to coordinate day-to-day work, drafting circumcision guidelines for health practitioners and using these to train service providers. The two bodies also approved the implementation of a situation analysis of MC in Tanzania. MC demonstration sites have been set up and are operational in the eight regions: Kagera, Mwanza, Mara, Shinyanga, Tabora, Iringa, Mbeya, and Rukwa for initial scale up process. MC scale up in Tanzania involved different stakeholders at national, regional, district and community levels. This called for clarity of leadership and partnerships to ensure success and sustainability of the programme from national to local governments. Partnerships facilitated advocacy for the scale up of MC for HIV-prevention, and brought resources, knowledge and experience from other programme areas.

At national level the role of partnerships has been realised in the working of the multi-stakeholders on MC national technical working group. These include bilateral agencies, international and national NGOs, the private health care providers, and professional bodies. At the regional and local government levels other partners relevant for advocacy, political support and ensuring an equitable MC programme include civil society groups, youth and women's interests and pressure groups; community and cultural gatekeepers.

Different partners will need to address different aspects of the essential component based on their respective mandates and strengths. For partnerships to attain optimum programme benefits it will be necessary to clarify each partner's role. An institutional responsibility is one of the key elements of a national MC policy [9]. In Tanzanian for example, the lobby and advocacy group led the advocacy and mobilisation campaigns, donor community funded the situation analysis study through the government of Tanzania. Donors also funded the development of national strategy for scaling up MC for HIV prevention and MC demonstration sites. Researchers conducted the situation analysis study and are conducting operational research studies on MC. NGOs and other partners are implementing demonstration sites.

Therefore, the introduction and scaling up of MC services requires actions from several key stakeholders. In Tanzania the technical working group ensured the active participation of policymakers, donors, advocacy groups, researchers and implementing partners. It was this partnership of various stakeholders of MC that was crucial in setting into motion the drive to roll-out MC services in Tanzania [15]. Furthermore, the importance of taking on board traditional gatekeepers in MC scale up efforts has been emphasised in another study in Tanzania [16].

The existing health system in Tanzania separates responsibility for addressing reproductive health and HIV-related concerns. A first step in integration is to adopt policies that support integrated and client-focused services. It can be argued that integrated approaches to deliver MC services with other essential HIV and sexual health services are most likely to be sustainable in the long term. However, vertical, stand alone programmes that provide the recommended minimum package of services may be useful in the short term to expand access to safe MC services and train providers in standardised procedures, especially where demand is high and health systems are weak.

Considering that MC services will be provided at different levels from the health units to the national level, it will be necessary to have in place accurate, timely and comprehensive data and information for planning purposes and ensuring quality of services. This also calls for identifying key input, process, outcome, output and impact indicators which will assist programme managers at all levels to oversee the quality of services and provide targets for supervisory activities. Countries should establish systems for monitoring the safety and quality of MC services. The recommended MC services standards, criteria and minimum package of services to be provided and the roles and responsibilities of national and district programme managers and facility managers and staff for implementing safe services of high quality; and guidance on the planning of a national quality assurance programme have been outlined in the WHO guide [17]. More importantly, a minimum package of services should be established in line with the WHO minimum package [9] and made available in all facilities providing MC. It should be discussed and agreed upon as part of the development of MC policy and strategy.

The introduction of MC on a scale that would generate population-level health outcomes requires broad societal acceptance. Consequently, wide support in civil society and target population in particular is necessary. Target population has been specified as one of key elements of a national MC policy [9]. Advocacy plays a crucial role from the outset of any programme for the introduction or scale up of MC, building support for key decisions on policy and MC activities and encouraging high-level leadership. There is a need for advocacy throughout the scale up process, overlapping with aspects of communication on social change. Social change communication may be used to foster a favourable political environment, generate acceptance and demand, and support services of high quality for MC. The eight steps to effective communication on MC have been outlined in the WHO & UNAIDS publication [9].

## Conclusions

Suffice it to conclude that findings of this study have shown that for successful implementation of the MC programme, first and foremost we need a favourable policy environment, then the necessary support at different levels of authority and the meaningful involvement of all stakeholders in the whole process.

Secondly, although there is no stand alone (formal/specific) policy on MC in Tanzania, basic elements of enabling policy environment at national, regional, district and community levels are in place (i.e. existence of effective regulations and enabling HIV/AIDS prevention policies which are sensitive to context: political, cultural and practical) and can be adapted for implementation of MC scale up programme.

## Competing interests

The authors declare that they have no competing interests.

## Authors' contributions

JRM, MW, JFM and JC, designed the study. JRM, FM, MW, JFM collected data. JRM and GM analysed data. JRM drafted the manuscript. All authors provided editorial inputs and approved the final version of the manuscript.

## Pre-publication history

The pre-publication history for this paper can be accessed here:

http://www.biomedcentral.com/1471-2458/11/506/prepub
